# Ultrasound-based quantitative tools in predicting renal involvement in asymptomatic primary hyperparathyroidism

**DOI:** 10.1007/s40618-023-02284-0

**Published:** 2024-01-27

**Authors:** B. Candemir, F. Cuce, S. Akın, N. E. Gulcelik

**Affiliations:** 1 Gulhane Faculty of Medicine, Department of Endocrinology and Metabolism, University of Health Sciences, Keçiören, 06010 Ankara, Turkey; 2 Gulhane Faculty of Medicine, Department of Radiology, University of Health Sciences, Ankara, Turkey

**Keywords:** Acoustic radiation force impulse, Elastography, Primary hyperparathyroidism, Renal resistive index, Shear wave velocity, Ultrasonography

## Abstract

**Purpose:**

Asymptomatic primary hyperparathyroidism (aPHPT) has been recognized as a condition that can lead to renal complications. Timely identification of prognostic indicators for renal impairment holds the potential to facilitate proactive monitoring and treatment strategies in these patients. This study aims to investigate the utility of acoustic radiation force impulse (ARFI) imaging and renal resistive index (RRI), in identifying renal parenchymal and vascular changes in patients with aPHPT.

**Methods:**

Forty-two patients with aPHPT and 42 controls matched for age, sex, and body mass index were included in the study. The presence of renovascular changes was evaluated by RRI measurement with Doppler ultrasonography, and the presence of renal parenchymal involvement was evaluated by ARFI quantification, given as shear wave velocity (SWV).

**Results:**

In aPHPT patients, both the mean RRI and mean SWV values exhibited substantial elevation compared to the control group (*P* < 0.001 for both). Significant associations were observed between SWV values and serum calcium, parathyroid hormone (PTH), and adenoma size within the patient group (*P* < 0.001, *P* < 0.001, *P* = 0.016, respectively). Similarly, the mean RRI demonstrated positive correlations with serum calcium and PTH levels in the patient group (*P*< 0.001, *P* = 0.011, respectively). Multivariate linear regression analysis underscored the connection between mean RRI and mean SWV values with serum calcium levels within the patient group. In addition, serum PTH levels affected mean SWV positively and significantly.

**Conclusion:**

The use of ARFI imaging and RRI measurements appears to hold potential in identifying renal involvement in patients with aPHPT.

## Introduction

Primary hyperparathyroidism (PHPT) is a systemic disorder characterized by inappropriately elevated parathyroid hormone (PTH) in the presence of high or normal serum calcium, and it is the most common cause of hypercalcemia [1]. Patients with PHPT may have renal involvement characterized by decreased renal function, hypercalciuria, nephrolithiasis, and/or nephrocalcinosis [1]. The current guidelines for asymptomatic PHPT (aPHPT) suggest surgery in the presence of renal end-organ damage, such as an estimated glomerular filtration rate (eGFR) below 60 ml/min/1.73 m^2^, significant hypercalciuria, and the presence of nephrolithiasis and/or nephrocalcinosis [2, 3]. Although most of the studies demonstrate improvement in renal functions with parathyroidectomy others fail to demonstrate an improvement, especially in patients with decreased GFR [4, 5]. The early determination of renal damage may improve renal surveillance in these patients leading to more active follow-up and treatment approaches.

Renal ultrasonography (US) is a cost-effective and easily accessible radiological method that can provide both morphological and functional data about kidneys, even in the absence of any symptoms [2, 6]. In a conventional US examination, grayscale imaging is used to detect the presence of nephrolithiasis and/or nephrocalcinosis, as well as to assess morphological features such as kidney size, cortical thickness, and echogenicity [7]. Doppler ultrasound, on the other hand, is a valuable tool for the diagnosis and management of renal vascular pathologies [8]. Renal resistive index (RRI), a non-invasive measure obtained from Doppler US, is used to evaluate pathological changes in the kidneys [9]. Increased RRI indicates increased intrarenal arterial resistance and the presence of albuminuria, and has been considered to predict the progression of chronic kidney disease (CKD) in patients with diabetes mellitus (DM) and hypertension [8, 9]. However, to our knowledge, the role of the RRI in predicting renal impairment in patients with PHPT has still not been investigated.

Elasticity imaging methods are used to distinguish between structures with different mechanical properties by applying stress to the tissue [10]. Acoustic radiation force impulse (ARFI) is an imaging method that can be used to measure the elastic properties of various soft tissues [11, 12]. In the ARFI method, a quantitative assessment of elasticity value can be achieved by the measurement of shear wave velocity (SWV). Some studies have shown that SWV measurement of the renal cortex is associated with various renal parenchymal diseases and parenchymal fibrosis, and the SWV values correlated with serum creatinine and eGFR [11, 13, 14]. Stock et al. have found a significant positive correlation between the mean SWV values and the grade of fibrosis in transplant kidneys [15].

Ascertaining the risk of target organ damage in an early phase is crucial for the management of patients with aPHPT. Therefore, embracing more refined and detailed methods, such as renal ARFI elastography measurements and RRI in patients with PHPT may empower healthcare practitioners to provide early interventions, personalized treatment strategies, and improved prognostic insights. This study aims to investigate the effectiveness of renal ARFI elastography and RRI measurements in identifying renal parenchymal and vascular changes in hypercalcemic patients with aPHPT who exhibit seemingly no apparent renal dysfunction with a comparative analysis against a control group.

## Materials and methods

### Study population

Between May 2021 and May 2022, 85 consecutive hypercalcemic patients with aPHPT and 2125 controls who visited our outpatient clinic were prospectively evaluated. The control group consisted of individuals who visited our outpatient clinic for reasons other than parathyroid pathology and healthy volunteers who applied for routine check-ups. After applying the exclusion criteria, renal US examination was performed on controls (*n* = 57) and patients (*n* = 63) who agreed to participate in the study. Those with solitary kidney, renal cyst, stone, renal mass, hydronephrosis, or renal artery stenosis were excluded from the study. Finally, 42 hypercalcemic patients with aPHPT and 42 controls matched for age, sex, and body mass index (BMI, kg/m^2^) were included. Exclusion criteria were as follows: age less than 18 years, renal transplantation, renal failure (eGFR < 60 ml/min/1.73 m^2^), urinary albumin/creatinine ratio > 30 mcg/mg, poorly controlled DM, uncontrolled hypertension, chronic liver disease, cardiovascular diseases (myocardial infarction, ischemic stroke, heart failure, atrial fibrillation or coronary revascularization), thyroid function disorders, malignancy, use of angiotensin-converting enzyme inhibitors/angiotensin receptor blockers, proton pump inhibitors, calcium or vitamin D supplements, and obese subjects with a renal depth of more than 8 cm by the skin surface. The diagnosis of PHPT was made by conventional clinical and laboratory criteria, including a history of at least 6 months of hypercalcemia without evidence of a nonparathyroid etiology. Patients with aPHPT were diagnosed and evaluated according to the current guidelines [2, 3, 16]. Sociodemographic data such as age, gender, and, smoking, clinical information, such as BMI, systolic blood pressure (SBP), diastolic blood pressure (DBP), comorbidities, and medications of all the individuals were obtained from anamneses, medical records, and the national health database. Several serum biochemical parameters, such as serum total calcium, phosphorus, albumin, glucose, creatinine, and 25-hydroxyvitamin D levels were measured with standard laboratory methods in all participants. In addition, the serum PTH and 24-h urinary calcium levels were also assessed in all patients with aPHPT. Biochemical measurements were performed on the same day of the radiological examinations or 1 day before.

### Radiological evaluation

All patients had at least an 8-h fast and a 15-min rest before undergoing the US examination. Radiological examinations were performed using an S3000 Acuson system (Siemens Healthcare) equipped with an ARFI function (Virtual Touch Tissue Quantification package, Siemens Healthcare, Erlangen, Germany) and a 4 to 6-MHz convex probe. All the radiologic examinations were performed by the same radiologist (F.C) who had at least 15 years of experience and was blinded to the clinical and biochemical data of both groups. All the subjects were placed in the left and right lateral decubitus positions for imaging of the right and left kidneys, respectively. Grey-scale B-mode US evaluation was first performed to assess kidney sizes, cortical thickness, and parenchyma echogenicity. Kidney length was measured in the coronal plane from the upper pole to the lower pole of the kidney. Cortical thickness was recorded as the distance from the medial section of the renal medullary pyramid base to the renal capsule.

Acoustic radiation force impulse imaging methods generate mechanical excitation using short-duration acoustic pulses in a region of interest (ROI), producing shear waves that spread away from the targeted ROI [10, 17]. After ARFI induction, softer (elastic) tissues are displaced more than stiffer (non-elastic) tissues, and the displacement time is prolonged. This results in greater SWV measurements in stiffer tissues [12]. Therefore, the SWV is considered mathematically proportional to tissue stiffness [18]. In virtual touch quantification, measurements were obtained after a region of interest (ROI) placement with dimensions of 10 × 6 mm. During the ultrasound, the ROI cursor was placed over the whole three main cortical regions of the kidney, including the upper and lower poles and middle cortex. The kidney capsule and collecting system were excluded. Six valid SWVs (expressed in meters per second, m/s) were obtained in each kidney and the cortical stiffness (CS) for each kidney was calculated as the mean of six valid measurements. The mean CS was the average of 12 SWVs in each participant. The measurement was repeated if an invalid measurement (expressed as X.XX m/s) was obtained [18, 19]. All the SWVs were performed, while the patients were holding their breath at the end of expiration. For evaluation of the RRI, peak systolic velocity (PSV, centimeters per second) and end-diastolic velocity (EDV, centimeters per second) were measured in interlobular arteries when the Doppler angle was 30–60° in the right and left kidneys. The RRI value was automatically calculated with PSV–EDV/PSV formula. RRI values were obtained from three different interlobar arteries and mean RRIs were presented as averages of three valid measurements in each kidney. The mean examination time for US imaging was approximately 20–30 min for each subject. To investigate the intraobserver reliability, repeated SWV measurements were performed on 20 consecutive participants by the radiologist on 2 different days.

### Statistical analyses

Statistical analysis was performed by IBM SPSS Statistics for Windows (Version 25.0. Armonk, NY). Continuous variables were investigated using the Kolmogorov–Smirnov test to determine whether or not they are normally distributed. The Student’s *t*-test was used for comparisons of normally distributed variables, and the Mann–Whitney *U* test was used for non-normally distributed variables. Continuous variables were expressed as means ± standard deviation and medians (interquartile range) according to distribution pattern. Categorical variables were presented as numbers and percentages and were compared with the Chi-square test. The correlations between the mean SWV and several variables (e.g., age, serum calcium, PTH, eGFR, and adenoma size) in the patients with asymptomatic PHPT were analyzed with Pearson’s test. The correlations between the mean RRI and the same parameters were also done in the same method. The presence of outliers among the numerical values included in the analysis was evaluated with Residual Statistics (standardized residuals value accepted between − 3,29 and + 3,29; Cook’s distance value accepted < 0.5 for all parameters). No outliers were found in any of the variables. Multivariate linear regression models with enter methods were used to identify independent predictors of RRI and SWV. The multicollinearity between the variables included in the multivariate linear regression analysis was analyzed by Pearson correlation analysis, tolerance values, variance inflation factor (VIF) values, and the condition index. There was no multicollinearity among any of the independent variables (Pearson correlation coefficient (*r*) < 0.70; tolerance > 0.25 and variance inflation factor < 4; condition number < 10; for all parameters. In addition, scatter dot plots were used to test whether there was a linear correlation between the dependent variable and the numerical independent variables for multivariate linear regression analysis. There was linearity between all variables and the dependent variable. All statistical analyses were performed two-sided and a *P*-value less than 0.05 was considered as statistically significant.

## Results

### The baseline characteristics of the study population

**Fig. 1:**
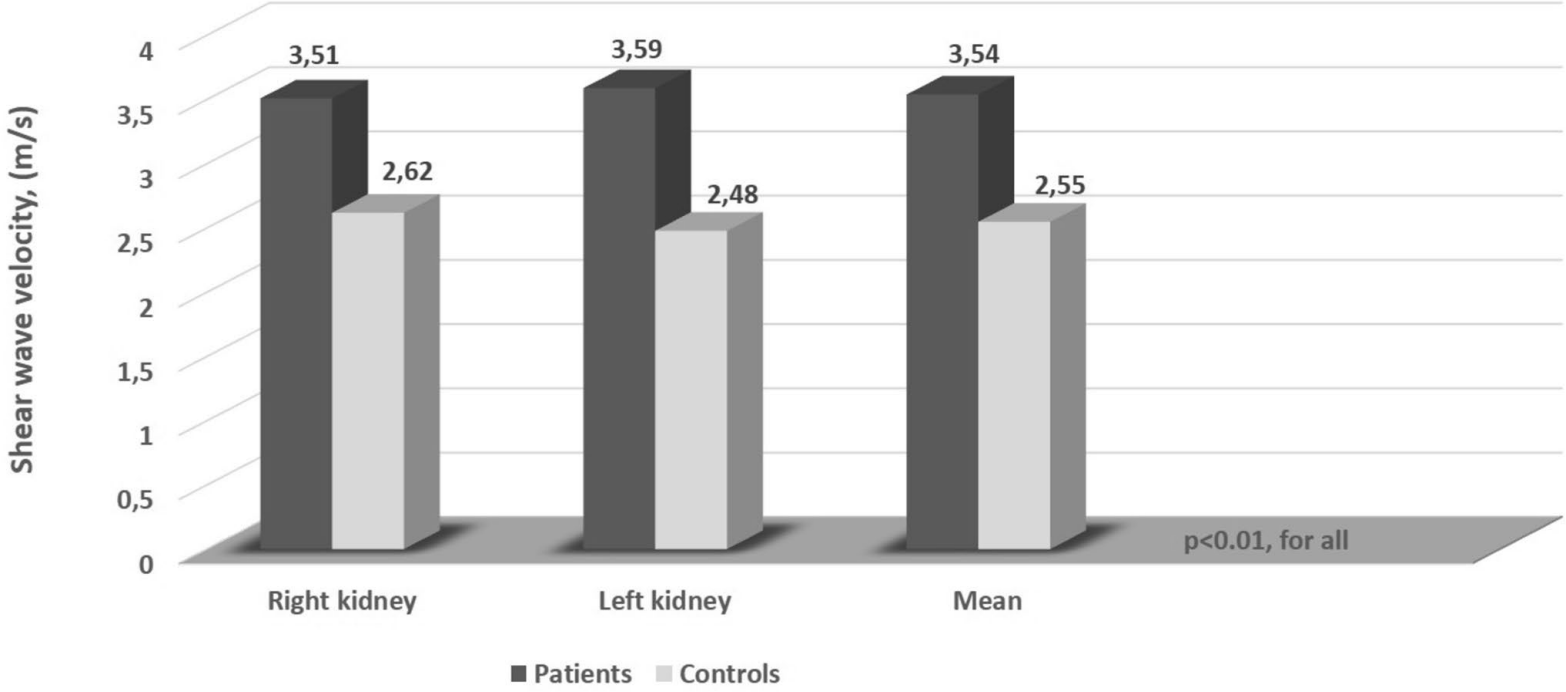
Comparison of the shear wave velocity values in patients with asymptomatic primary hyperparathyroidism and controls

**Fig. 2:**
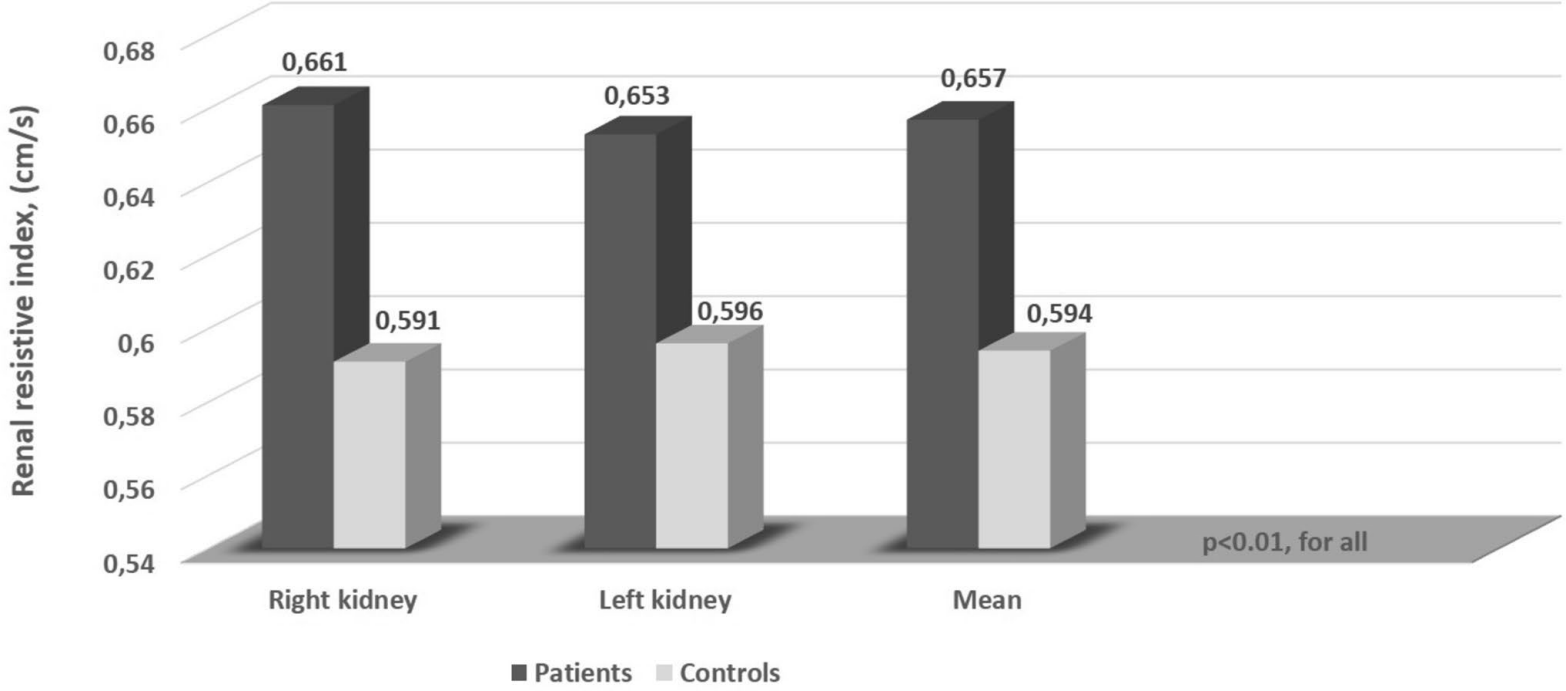
Comparison of the renal resistive index in in patients with asymptomatic primary hyperparathyroidism and controls

A total of 42 patients with aPHPT and 42 controls were included in the study. The mean age was 53.6 ± 9.4 years in the patient group and it was 53.6 ± 9.7 years in the control group (*P* = 0.97). There was no significant difference between both groups in terms of smoking, BMI, comorbidities (hypertension, dyslipidemia), and baseline biochemical values (except serum calcium and phosphorus). In the patient group, the mean renal SWV value was 3.54 ± 0.43 m/s, while in the control group, it was 2.55 ± 0.5 m/s (*P* < 0.001) (Fig. 1). The mean RRI value was higher in the patient group than in the control group (0.657 ± 0.04 cm/s vs. 0.594 ± 0.04, *P* < 0.001) (Fig. 2). The baseline clinical and biochemical characteristics of the study population are shown in Table 1.

**Table 1:** Baseline characteristics of the study population

	Patients (*n* = 42)	Controls (*n* = 42)	*P*
Age, years	53.6 ± 9.4	53.6 ± 9.7	0.973
Gender male, *n* (%)	3 (7.1)	4 (9.59)	1
Smoking, *n* (%)	6 (14.3)	7 (16.7)	0.763
DM, *n* (%)	12 (28.6)	13 (31)	0.811
Hypertension, *n* (%)	18 (42.9)	21 (50)	0.512
Dyslipidemia, *n* (%)	11 (26.2)	8 (19)	0.434
Systolic BP, (mmHg)	123.16 ± 10.94	118.8 ± 17.31	0.172
Diastolic BP, (mmHg)	77.9 ± 7.41	75.3 ± 13.02	0.264
BMI, (kg/m^2^)	30.57 ± 5.2	29.34 ± 4.64	0.259
Hemoglobin, (g/dl)	13.55 (13–14.1)	13.45 (12.77–14.4)	0.764
Glucose, (mg/dl)	92.07 ± 8.37	91.92 ± 9.31	0.941
Calcium, (mg/dl)	11.29 ± 0.65	9.5 ± 0.48	** < 0.001**
Phosphorus, (mg/dl)	2.77 ± 0.51	3.55 ± 0.61	** < 0.001**
Creatinine, (mg/dl)	0.79 ± 0.11	0.77 ± 0.09	0.453
GFR, (ml/min/1.73 m^2^)	84.18 ± 13.25	88.14 ± 14.55	0.197
PTH, (pg/ml)	159.94 ± 40.56	–	–
Urinary calcium, (mg/d)	356.20 ± 156.84	–	–
25 (OH) D, (ng/ml)	24 (11.76–32.82)	24.4 (11.43–35.58)	0.893
HbA1c, (%)	7.18 ± 0.64	7.27 ± 0.59	0.711
Right RRI, (cm/s)	0.661 ± 0.04	0.591 ± 0.04	** < 0.001**
Left RRI, (cm/s)	0.653 ± 0.05	0.596 ± 0.05	** < 0.001**
Mean RRI, (cm/s)	0.657 ± 0.04	0.594 ± 0.04	** < 0.001**
Right SWV, (m/s)	3.51 ± 0.42	2.62 ± 0.64	** < 0.001**
Left SWV, (m/s)	3.59 ± 0.5	2.48 ± 0.51	** < 0.001**
Mean SWV, (m/s)	3.54 ± 0.43	2.55 ± 0.5	** < 0.001**
Right CT, (mm)	16.66 ± 2.98	16.73 ± 2.88	1
Left CT, (mm)	16.88 ± 2.75	17.07 ± 3.14	0.780
Right kidney length, (mm)	109.24 ± 9.92	107.48 ± 8.63	0.389
Left kidney length, (mm)	112.14 ± 9.83	109.31 ± 8.45	0.160
Adenoma size, (mm)	15.24 ± 4.09	–	–

*BMI* body mass index, *BP* blood pressure, *CT* cortical thickness, *DM* diabetes mellitus, *GFR* glomerular filtration rate, *HbA1c* glycolized hemoglobin, *PTH* parathyroid hormone, *RRI* renal resistive index, *SWV* shear wave velocity, *25 (OH) D* 25-hydroxy vitamin D

Results are expressed as mean ± standard deviation or median (interquartile range) according to the distribution pattern. Significant *P* values are in bold

### Correlation analysis between shear wave velocity, renal resistive index, and several parameters of the study population

In the patient group, there was a significant correlation between serum calcium, PTH, and adenoma size with the SWV values (*r* = 0.573; *P* < 0.001, *r* = 0.634; *P* < 0.001, *r* = 0.412; *P* = 0.01, respectively). There was no significant correlation between the average SWV measurements and eGFR in the patient group (*P* = 0.726). The mean RRI value was positively correlated with serum calcium and PTH levels in the patient group (*r* = 0.544; *P* < 0.001, *r* = 0.389; *P* = 0.01, respectively) (Table 2). There was no significant correlation between the mean RRI values and the BMI, eGFR, SBP, and DBP values in the patient group (*P* > 0.05, for all). There was no statistically significant correlation between age, serum calcium, serum phosphorus level, BMI, SBP, and DBP values with both mean SWV and mean RRI values, in the control group (*P* > 0.05, for all).

**Table 2:** Correlation analysis between several parameters and radiological examination in the patient group

		Age	Calcium	Phosphorus	PTH	eGFR	Urinary calcium	Duration of hypercalcemia	Adenoma size
Mean SWV	*r*	− 0.113	0.573	− 0.073	0.634	− 0.056	0.018	− 0.057	0.412
	*P*	0.475	** < 0.001**	0.648	** < 0.001**	0.726	0.913	0.751	**0.016**
Mean RRI	*r*	0.286	0.544	− 0.116	0.389	− 0.133	− 0.288	− 0.066	− 0.116
	*P*	0.066	** < 0.001**	0.464	**0.011**	0.403	0.068	0.71	0.512

*eGFR* estimated glomerular filtration rate, *PTH* parathyroid hormone, *RRI* renal resistive index, *SWV* shear wave velocity, *25 (OH) D* 25-hydroxy vitamin D

Significant *P* values are in bold

### Multivariate linear regression analysis for independent variables of shear wave velocity and renal resistive index in the patient group

**Fig. 3:**
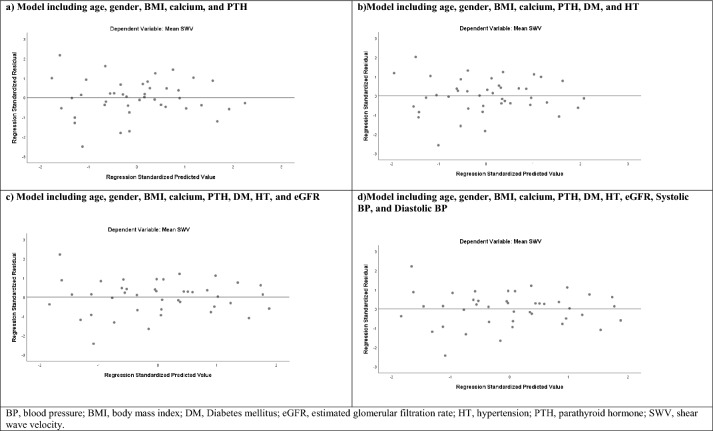
Development of a linear model to predict shear wave velocity

**Fig. 4:**
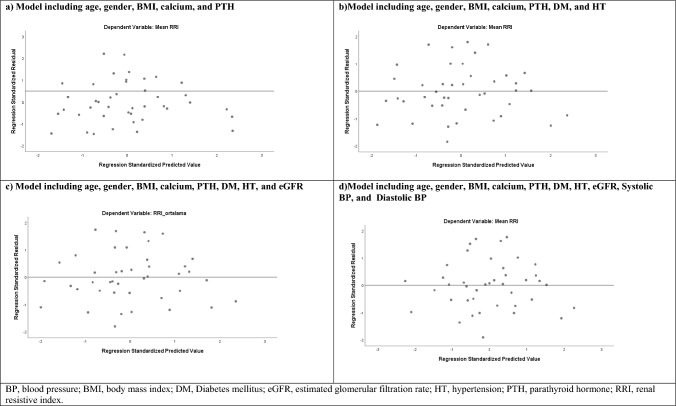
Development of a linear model to predict renal resistive index

Multivariate linear regression analyses with SWV as a dependent variable were applied in a final model that included age, gender, BMI, serum calcium, PTH, DM, HT, eGFR, SBP, and DBP, showing for serum calcium and PTH levels a significant association with SWV. In addition, multivariate linear regression analyses with RRI as a dependent variable were applied in a final model that included the same parameters, showing serum calcium a significant association with RRI. Figures 3a, b, c, d and 4a, b, c, d show model development and the final model with linear prediction of SWV and RRI.

The independent variables included in the models explained about 47% of the variation in SWV as determined by multivariate linear regression analysis (Adjusted R^2^, 0.486 for model 1; adjusted R^2^, 0.470 for model 2; adjusted R^2^, 0.467 for model 3; adjusted R^2^, 0.454 for model 4; *P* < 0.05, for all). The mean SWV was significantly associated with serum calcium level (beta: 0.379, *P* = 0.01, for model 1; beta: 0.387, *P* = 0.01, for model 2; beta: 0.408, *P* = 0.01, for model 3; beta: 0.343, *P* = 0.04, for model 4). In addition, PTH levels exhibited a statistically significant and positive effect on SWV in all models (beta: 0.357, *P* = 0.03, for model 1; beta: 0.384, *P* = 0.02, for model 2; beta: 0.360, *P* = 0.03, for model 3; beta: 0.391, *P* = 0.02, for model 4) (Table 3).

**Table 3:** Multivariate linear regression analysis of independent predictors of SWV in the patient group

	Multivariate linear regression analysis*						
	B	Standard error	Standardized coefficients beta	%95 CI		*t*	*P*
				Lower	Upper		
Model 1							
Age	− 0.014	0.006	− 0.310	− 0.027	–0.001	–2.214	0.033
Calcium	0.252	0.097	0.379	0.054	0.449	2.587	0.014
PTH	0.004	0.002	0.357	0.000	0.007	2.260	0.030
Model 2							
Calcium	0.257	0.100	0.387	0.054	0.460	2.575	0.015
PTH	0.004	0.002	0.384	0.001	0.008	2.354	0.024
Model 3							
Calcium	0.271	0.101	0.408	0.065	0.477	2.675	0.012
PTH	0.004	0.002	0.360	0.000	0.007	2.175	0.037
Model 4							
Calcium	0.227	0.111	0.343	0.002	0.453	2.054	0.048
PTH	0.004	0.002	0.391	0.000	0.008	2.296	0.029

*Only variables with a *P*-value of < 0.05 were presented

Model 1: Age, gender, BMI, calcium, PTH

*R*: 0.740, R^2^: 0.548, adjusted *R*^2^: 0.486, *F*: 8.739, *P*: < 0.001, Durbin–Watson: 1.617

Model 2: Model 1 + DM + HT

*R*: 0.749, R^2^: 0.560, adjusted *R*^2^: 0.470, *F*:6.194, *P*: < 0.001, Durbin–Watson: 1.631

Model 3: Model 2 + eGFR

*R*: 0.756, R^2^: 0.571, adjusted *R*^2^: 0.467, *F*: 5.494, *P*: < 0.001, Durbin–Watson: 1.635

Model 4: Model 3 + Systolic BP + Diastolic BP

*R*: 0.766, R^2^: 0.587, adjusted *R*^2^: 0.454, *F*: 4.408, *P*: 0.001, Durbin–Watson: 1.706

*BP* blood pressure, *BMI* body mass index, *DM* Diabetes mellitus, *eGFR* estimated glomerular filtration rate; *HT* hypertension, *PTH* parathyroid hormone, *SWV* shear wave velocity

The multivariate linear regression analysis revealed that independent variables included in the models for mean RRI explained approximately 29% of the RRI variation. (Adjusted *R*^2^, 0.270 for model 1; adjusted *R*^2^, 0.317 for model 2; adjusted *R*^2^, 0.307 for model 3; adjusted *R*^2^, 0.282 for model 4; *P* < 0.05, for all). Serum calcium levels affected mean RRI positively and significantly in all models (beta: 0.456, *P* = 0.01, for model 1; beta: 0.484, *P* = 0.008, for model 2; beta: 0.50, *P* = 0.007, for model 3; beta: 0.51, *P* = 0.011, for model 4) (Table 4).

**Table 4:** Multivariate linear regression analysis of independent predictors of RRI in the patient group

Parameters	Multivariate linear regression analysis*						
	B	Standard error	Standardized coefficients beta	%95 CI		*t*	*P*
				Lower	Upper		
Model 1							
Calcium	0.032	0.012	0.456	0.007	0.057	2.615	0.013
Model 2							
Calcium	0.034	0.012	0.484	0.010	0.058	2.835	0.008
Model 3							
Calcium	0.035	0.012	0.503	0.010	0.060	2.894	0.007
Model 4							
Calcium	0.036	0.013	0.517	0.009	0.063	2.703	0.011

*Only variables with a *P*-value of < 0.05 were presented

Model 1: Age, gender, BMI, calcium, PTH

*R*: 0.599, *R*^2^: 0.359, adjusted *R*^2^: 0.270, *F*: 4.038, *P*: 0.005, Durbin–Watson: 1.764

Model 2: Model 1 + DM + HT

*R*: 0.658, *R*^2^: 0.433, adjusted *R*^2^: 0.317, *F*: 3.715, *P*: 0.004, Durbin–Watson: 1.737

Model 3: Model 2 + eGFR

*R*: 0.665, *R*^2^: 0.443, adjusted *R*^2^: 0.307, *F*: 3.274, *P*: 0.007, Durbin–Watson: 1.756

Model 4: Model 3 + Systolic BP + Diastolic BP

*R*: 0.676, *R*^2^: 0.457, adjusted *R*^2^: 0.282, *F*: 2.609, *P*: 0.02, Durbin–Watson: 1.728

*BP* blood pressure, *BMI* body mass index, *DM* Diabetes mellitus, *eGFR* estimated glomerular filtration rate, *HT* hypertension, *PTH* parathyroid hormone, *RRI* renal resistive index

## Discussion

In the present study, we observed that mean renal SWV values and mean RRI were significantly higher in aPHPT patients compared to the control group. This disparity suggests a discernible deterioration in renal parenchymal elasticity and increased renal vascular resistance within the aPHPT patient cohort. There are few studies investigating some biochemical markers that predict the presence of mild and subclinical renal damage in PHPT [20]. However, to the best of our knowledge, this is the first study that investigates comprehensively renal parenchymal architectural changes and renal vascular involvement using several quantitative imaging tools in patients with aPHPT. Renal involvement is one of the most significant complications of primary hyperparathyroidism and is related to disease progression [2, 6]. Among the most frequent complications in PHPT are nephrolithiasis and nephrocalcinosis, both of which have the potential to lead to impaired renal function [6]. It was shown that 35.5% of asymptomatic patients with PHPT had nephrolithiasis [21]. Current guidelines suggest that US examination should be used as an initial imaging test in the detection of the presence of renal involvement in patients with PHPT [2, 6, 22]. However, focusing only on nephrolithiasis and nephrocalcinosis lacks the necessary precision and thoroughness required to accurately gauge early signs of renal complications in these patients. As the pathogenesis of renal manifestations in PHPT remains inadequately understood, it poses significant challenges in the diagnosis and timely management of patients with early-stage renal complications [23, 24]. Novel diagnostic modalities investigating renal parenchymal and vascular disease may provide valuable insights into the timely detection and management of these patients.

Acoustic radiation force impulse imaging is a useful method that can be used to predict several cortical pathological changes associated with renal functions [19, 25]. SWV values assessed through the ARFI method displayed correlations with eGFR in certain investigations probing the interplay between renal function and stiffness of the renal parenchyma [14, 25]. It was shown that the PTH receptor is expressed in renal glomerular endothelial and proximal tubule cells. High PTH exposure leads to morphological changes, including an expanded endoplasmic reticulum and increased vacuolization of mitochondria in the endothelium. Furthermore, PTH promotes endothelial-to-mesenchymal transition in the glomeruli, and in this way accelerates the progression of renal fibrosis [26]. Based on these data, it is conceivable that ARFI imaging holds promise as a valuable tool in the evaluation of renal parenchymal damage and/or renal functions in PHPT patients. In the present study, we found that the mean SWV values were significantly correlated with serum calcium and PTH levels. Although the majority of our patients exhibited hypercalciuria, it does not demonstrate a discernible correlation with SWV values. Our results are in line with other studies that similarly fail to establish a definitive connection between hypercalciuria and renal complications [24]. Although hypercalciuria is well known to be a risk factor for renal complications such as kidney stones, there are other risk factors such as hypomagnesuria and genetic factors [27]. In addition, we may have obtained these results because patients with nephrolithiasis/nephrocalcinosis were excluded in our study. Consequently, we may infer that hypercalciuria alone does not constitute a solitary factor in the pathogenesis of renal parenchymal damage. These findings suggest that hypercalcemia and elevated PTH levels are independent predictors of increased SWV and may play a role in the development of renal parenchymal damage.

Various studies investigating the factors affecting SWV values have reported associations with age, gender and, eGFR [25, 28]. It was shown that SWV values were significantly correlated with age and gender but not with BMI, in healthy controls [19]. In the multivariate linear regression analysis, we found that age, gender, SBP, DBP, eGFR, BMI, serum phosphorus level, and urinary calcium excretion did not have a significant effect on the mean SWV. Contrary to other studies, our lack of finding a relationship between eGFR and mean SWV values may be attributed to the normal eGFR values in our patient population, suggesting that SWV may be an indicator of early-stage damage in these patients [14]. In addition, we also found that the presence of DM was not an independent predictor of the SWV values in patients with aPHPT. This situation could be due to the absence of any participants with diabetic nephropathy affecting SWV values. After all, our findings demonstrated increased renal stiffness which is related to hypercalcemia and elevated PTH levels in patients with aPHPT.

The renal resistive index is a non-invasive and reproducible tool to assess intrarenal perfusion and renal vascular resistance [8, 29]. In one study, it was suggested that in addition to glomeruli, renal and intrarenal vascular endothelium are also affected by atherosclerosis in diabetic patients [9]. Doi et al. demonstrated that increased RRI was independently associated with cardiovascular and renal outcomes, such as death, myocardial infarction, stroke, and requiring hemodialysis in a follow-up study of patients with essential hypertension [30]. In the present study, the mean RRI was significantly positively correlated with serum calcium and PTH levels but it was not correlated with urinary calcium excretion. In multivariate linear regression analysis, we showed that only serum calcium level had a significant effect on mean RRI when different models were performed. Considering the established links between atherosclerotic disease and PHPT, RRI represents a straightforward and cost-effective method that could offer valuable insights into predicting complications related to renovascular involvement in asymptomatic PHPT patients.

Various studies investigating factors influencing RRI have yielded different results. It was shown that age, eGFR, HbA1c, DBP, and presence of DM were significantly associated with increased RRI [9, 31, 32]. In one study, eGFR was not identified as an independent risk factor for increased RRI in type 2 diabetic patients with preserved renal functions [9]. In the present study, we found that only serum calcium level was independently associated with RRI in the patient group. Our analyses revealed that the presence of DM was not an independent risk factor for increased RRI and this can be explained by the inclusion of only DM patients without diabetic nephropathy. The absence of an association between eGFR and RRI can be attributed to the possibility that the damage can be detected before a visible decrease in eGFR becomes apparent. Our results suggest that hypercalcemia can lead to adverse renovascular outcomes, as well as, renal parenchymal damage in patients with aPHPT, even if there is no prominent loss of renal function.

Although both mean SWV and RRI values were increased in the patient group compared to the controls, we observed no correlation between the mean SWV values and the RRI, in line with a previous study [15]. It is plausible that renal SWV and RRI values are potentially independent variables, as they assess distinct mechanisms of the disease. Consequently, the strategies employed for the prevention or management of renal complications of PHPT might necessitate a tailored approach for addressing each individual concern.

Our study has several limitations. First, the cross-sectional design of the study does not rule out the possibility of unintentional confounding by unknown factors, although we attempted to address this concern through strict exclusion criteria. Second, this study was conducted at a single center with a relatively small sample size. However, a focused approach in a single center facilitates standardized data collection and consistency in data interpretation. The performance of ultrasound by a single physician may also reduce interobserver variability. Third, the absence of subjects with symptomatic PHPT and/or decreased eGFR might impact the broader applicability of the results. Lastly, the lack of histopathologic data constitutes another limitation of our study.

In conclusion, we showed that both the mean SWV values evaluated by ARFI imaging and the mean RRI values in aPHPT patients with no apparent renal dysfunction were significantly increased compared to the control group. Our findings support that renal parenchymal and vascular adverse outcomes begin before renal functions are affected in these patients. The use of SWV and RRI in aPHPT patients can aid in the early diagnosis of renal involvement, allowing for timely interventions to prevent or mitigate renal complications. Monitoring SWV and RRI over time can provide insights into the progression of renal alterations and help guide treatment decisions. Future research endeavors will pave the way for a more comprehensive understanding of the role of SWV and RRI in determining renal involvement in aPHPT.

## Data Availability

The datasets generated during the current study are not publicly available but are available from the corresponding author on reasonable request.

## References

[1] Bilezikian JP, Brandi ML, Rubin M, Silverberg SJ (2005). Primary hyperparathyroidism: new concepts in clinical, densitometric and biochemical features. J Intern Med.

[2] Bilezikian JP, Brandi ML, Eastell R (2014). Guidelines for the management of asymptomatic primary hyperparathyroidism: summary statement from the fourth international workshop. J Clin Endocrinol Metab.

[3] Bilezikian JP, Khan AA, Silverberg SJ (2022). Evaluation and management of primary hyperparathyroidism: summary statement and guidelines from the fifth international workshop. J Bone Miner Res.

[4] Agrawal K, Arya AK, Sood A (2021). A detailed appraisal of renal manifestations in primary hyperparathyroidism from Indian PHPT registry: Before and after curative parathyroidectomy. Clin Endocrinol (Oxf).

[5] Tassone F, Guarnieri A, Castellano E, Baffoni C, Attanasio R, Borretta G (2015). Parathyroidectomy halts the deterioration of renal function in primary hyperparathyroidism. J Clin Endocrinol Metab.

[6] Rejnmark L, Vestergaard P, Mosekilde L (2011). Nephrolithiasis and renal calcifications in primary hyperparathyroidism. J Clin Endocrinol Metab.

[7] Manley JA, O'Neill WC (2001). How echogenic is echogenic? Quantitative acoustics of the renal cortex. Am J Kidney Dis.

[8] Viazzi F, Leoncini G, Derchi LE, Pontremoli R (2014). Ultrasound Doppler renal resistive index: a useful tool for the management of the hypertensive patient. J Hypertens.

[9] Hamano K, Nitta A, Ohtake T, Kobayashi S (2008). Associations of renal vascular resistance with albuminuria and other macroangiopathy in type 2 diabetic patients. Diabetes Care.

[10] Doherty JR, Trahey GE, Nightingale KR, Palmeri ML (2013). Acoustic radiation force elasticity imaging in diagnostic ultrasound. IEEE Trans Ultrason Ferroelectr Freq Control.

[11] Zaffanello M, Piacentini G, Bruno C, Brugnara M, Fanos V (2015). Renal elasticity quantification by acoustic radiation force impulse applied to the evaluation of kidney diseases: a review. J Investig Med.

[12] D'Onofrio M, Gallotti A, Mucelli RP (2010). Tissue quantification with acoustic radiation force impulse imaging: Measurement repeatability and normal values in the healthy liver. AJR Am J Roentgenol.

[13] Koc AS, Sumbul HE, Gülümsek E (2019). Increased renal cortical stiffness in patients with advanced diabetic kidney disease. Saudi J Kidney Dis Transpl Jan-Feb.

[14] He WY, Jin YJ, Wang WP, Li CL, Ji ZB, Yang C (2014). Tissue elasticity quantification by acoustic radiation force impulse for the assessment of renal allograft function. Ultrasound Med Biol.

[15] Stock KF, Klein BS, Vo Cong MT (2010). ARFI-based tissue elasticity quantification in comparison to histology for the diagnosis of renal transplant fibrosis. Clin Hemorheol Microcirc.

[16] Marcocci C, Bollerslev J, Khan AA, Shoback DM (2014). Medical management of primary hyperparathyroidism: proceedings of the fourth International Workshop on the Management of Asymptomatic Primary Hyperparathyroidism. J Clin Endocrinol Metab.

[17] Nightingale K, Soo MS, Nightingale R, Trahey G (2002). Acoustic radiation force impulse imaging: in vivo demonstration of clinical feasibility. Ultrasound Med Biol.

[18] Bob F, Bota S, Sporea I, Sirli R, Popescu A, Schiller A (2015). Relationship between the estimated glomerular filtration rate and kidney shear wave speed values assessed by acoustic radiation force impulse elastography: a pilot study. J Ultrasound Med.

[19] Goya C, Kilinc F, Hamidi C (2015). Acoustic radiation force impulse imaging for evaluation of renal parenchyma elasticity in diabetic nephropathy. AJR Am J Roentgenol.

[20] Cipriani C, Pepe J, Colangelo L (2019). Investigating subtle kidney injury in primary hyperparathyroidism by means of sensitive and specific biomarkers. Clin Endocrinol (Oxf).

[21] Cipriani C, Biamonte F, Costa AG (2015). Prevalence of kidney stones and vertebral fractures in primary hyperparathyroidism using imaging technology. J Clin Endocrinol Metab.

[22] Silverberg SJ, Clarke BL, Peacock M (2014). Current issues in the presentation of asymptomatic primary hyperparathyroidism: Proceedings of the Fourth International Workshop. J Clin Endocrinol Metab.

[23] Ejlsmark-Svensson H, Bislev LS, Rolighed L, Sikjaer T, Rejnmark L (2018). Predictors of renal function and calcifications in primary hyperparathyroidism: a nested case-control study. J Clin Endocrinol Metab.

[24] Frøkjaer VG, Mollerup CL (2002). Primary hyperparathyroidism: renal calcium excretion in patients with and without renal stone sisease before and after parathyroidectomy. World J Surg.

[25] Guo LH, Xu HX, Fu HJ, Peng A, Zhang YF, Liu LN (2013). Acoustic radiation force impulse imaging for noninvasive evaluation of renal parenchyma elasticity: preliminary findings. PLoS ONE.

[26] Wu M, Tang RN, Liu H, Ma KL, Lv LL, Liu BC (2014). Nuclear translocation of β-catenin mediates the parathyroid hormone-induced endothelial-to-mesenchymal transition in human renal glomerular endothelial cells. J Cell Biochem.

[27] El-Hajj Fuleihan G, Chakhtoura M, Cipriani C (2022). Classical and nonclassical manifestations of primary hyperparathyroidism. J Bone Miner Res.

[28] Bota S, Bob F, Sporea I, Şirli R, Popescu A (2015). Factors that influence kidney shear wave speed assessed by acoustic radiation force impulse elastography in patients without kidney pathology. Ultrasound Med Biol.

[29] Tublin ME, Bude RO, Platt JF (2003). Review. The resistive index in renal Doppler sonography: where do we stand. AJR Am J Roentgenol.

[30] Doi Y, Iwashima Y, Yoshihara F (2012). Renal resistive index and cardiovascular and renal outcomes in essential hypertension. Hypertension.

[31] Calabia J, Torguet P, Garcia I (2014). The relationship between renal resistive index, arterial stiffness, and atherosclerotic burden: the link between macrocirculation and microcirculation. J Clin Hypertens (Greenwich).

[32] Matsumoto N, Ishimura E, Taniwaki H (2000). Diabetes mellitus worsens intrarenal hemodynamic abnormalities in nondialyzed patients with chronic renal failure. Nephron.

